# Short-time work in Luxembourg: evidence from a firm survey

**DOI:** 10.1186/s12651-018-0247-7

**Published:** 2018-12-04

**Authors:** Konstantinos Efstathiou, Thomas Y. Mathä, Cindy Veiga, Ladislav Wintr

**Affiliations:** 1 Bruegel, Brussels, Belgium; 2 Banque centrale du Luxembourg, Luxembourg, Luxembourg

**Keywords:** Firms, Survey, Crisis, Short-time work, C25, J63, J68

## Abstract

We analyse the use of short-time work (STW) by Luxembourg firms during the years of economic and financial crisis (2008–2009) and the subsequent European sovereign debt crisis (2010–2013). The economic and financial crisis saw a surge in the number of firms using short-time work. We find that the likelihood that a firm applied for or used short-time work increases with demand volatility, the degree of firm-specific human capital and is higher for firms that cannot shift workers between establishments or that are more export oriented. Firms reported that 20–25% of jobs in short-time work were saved by this measure.

## Introduction

After a long period of sustained growth, Luxembourg was severely affected in the initial phase of the global economic and financial crisis in 2008–2009. During the recession, Luxembourg authorities introduced a broad range of labour market policies in order to cushion the effects of the recession on the labour market. The policy package included a mix of activation measures designed to increase job opportunities for the unemployed and improve the matching between labour supply and demand, passive income replacement measures for those who lost their jobs and other measures designed to support labour demand, such as loosening the eligibility criteria of short-time work arrangements (STWA) (see Table 6 in Appendix 1 for further details). During 2009–2014, the number of people involved in active labour market policies rose continuously from about 3100 to 5000 (ADEM 2015). The number of employees involved in short-time work rose very rapidly to unprecedented levels in 2008–2009 (nearly 10,000 people in spring 2009, i.e. 4.5% of total employees) and quickly receded, only to gain new momentum in 2011–2012 and remain at elevated levels compared to the pre-crisis period until 2015 (ADEM 2015; *Comité de conjoncture*).

This paper studies short-time work (STW) in Luxembourg during the crisis and analyses their evolution, determinants and effects.^1^ Short-time work is intended to address transitory shocks. Short-time work is designed to help firms limit costly redundancies, preserve firm-specific human capital and avoid hiring and training costs in the subsequent upswing. STW is normally of short duration (Arpaia et al. 2010) and was widely used in many EU countries, including Luxembourg, during the economic and financial crisis. In Luxembourg, the law sets a maximum limit to the reduction in hours worked per employee in order to share the burden of adjustment across a larger number of workers. To be able to participate in short-time work, firms and employees have to meet eligibility criteria.

We exploit a firm survey conducted by the Central Bank of Luxembourg at the end of 2014. The survey asked human resources managers and/or CEOs in Luxembourg detailed questions about their companies’ characteristics, how they were affected by the crisis during 2008–2009 and 2010–2013 and how shocks and changes in the economic environment led them to adjust labour, wages and prices. The survey also included a set of questions on short-time work. We analyse which firms applied for and used short-time work and what effect this had on firm employment, or put differently how many jobs may have been saved through STW.

The paper contributes to the literature by presenting the first analysis of STW in Luxembourg using micro data.^2^ It employs a unique firm-level survey that allows us to study the impact of shocks on firms’ decision to apply for STW Furthermore, we compare our survey-based results to results derived from macro data for Luxembourg. Studying STW in Luxembourg is interesting as it shows the impact of STW in a country with specific labour market institutions, such as high degree of wage rigidity and a relatively high level of employment protection. The use of STW might be particularly useful for firms that find it difficult or costly to adjust their labour costs or employment.

While short-time work is largely a sector-specific phenomenon, concentrated especially in the manufacturing sector, we find that the likelihood of a firm using short-time work generally increases with demand volatility, the share of workers with permanent contracts, the extent of firm-specific human capital, the degree of export orientation and with the inability to shift workers between establishments. Direct answers from human resources managers and and/or companies’ CEOs suggest that short-time work may have saved 20–25% of jobs involved corresponding to 2400 jobs in 2008–2009 and 921 jobs in 2010–2013 if extrapolated to the whole economy. This corresponds to 0.7% and 0.3% of employment in the respective sub-periods.

The remainder of the paper is organised as follows. Section 2 provides a brief overview of the STW literature. Section 3 reviews the rise of STW during the Great Recession in Luxembourg. Section 4 presents the dataset used for the empirical analysis. Section 5 presents the empirical estimation strategy regarding STW take-up while Sect. 6 discusses the results. Section 7 attempts to evaluate the number of jobs saved by STW and Sect. 8 concludes.

## Background

### Introduction and theoretical considerations

Short-time work programmes aim to avoid excessive layoffs in response to temporary fluctuations in demand. Given that labour is a quasi-fixed input of production, firms may engage in excessive layoffs in a context of demand volatility (Oi 1962). A temporary drop in demand requires short-term adjustment of inputs, which will eventually be reversed once demand recovers. In the short-run, if capital is fixed and labour is variable, then the latter input bears the entire adjustment burden. However, in practice labour may not be completely variable due to fixed costs of hiring, firing and training. These expenses need to be amortised over the course of the employment relationship and, thus, require a sizeable fall in demand and, in turn, in the value of the worker’s marginal product to justify a layoff on efficiency terms.

It follows that the size of the slump needed to make a separation efficient is increasing in the degree of fixity, which varies across types of workers and depends positively on the size of recruitment and training costs, and the expected length of the employment relationship (Oi 1962). Recent studies of the effect of short-time work schemes explore this argument in more detail. Arpaia et al. (2010) cite avoidance of dismissal costs and savings on recruitment and training costs as strong incentives for employers to participate in short-time work. Crimmann et al. (2010) also note that firms enrolling in such schemes must first assess the direct monetary costs associated with heavy workforce turnover.

A firm is more likely to accept training expenses if the resulting productivity gains are firm-specific (Oi 1962). As demonstrated by Hall and Lazear (1984), inefficient layoffs occur when the marginal product of the worker is higher within the firm than outside of the firm. Therefore, the firm decision to enrol in a short-time work programme depends on how much it has already invested in firm-specific human capital. Skilled, tenured and specialised employees are more costly to dismiss given firm-specific human capital loss (Crimmann et al. 2010; Arpaia et al. 2010). Along the same lines, Hijzen and Venn (2011) expect firms in manufacturing to be more inclined to resort to short-time work than firms in construction since their labour skills will be more firm-specific.

Short-time work arrangements can in principle promote efficient outcomes; however, they tend to give rise to complex effects due to their design and their interactions with other policies and labour market institutions. Burdett and Wright (1989) investigate how interactions between work-sharing compensation schemes and unemployment insurance tax incentives affect the efficiency of labour adjustments. They demonstrate that the absence of short-time work compensation leads to a bias in favour of layoffs, but its presence results in a distortion in hours and underemployment if the same tax parameters are imposed. The inefficiency often results from these two systems not being fully experience-rated. Working with a similar framework, Van Audenrode (1994) stresses that the combined effect of subsidised hours reduction and firing restrictions usually favours adjustment through hours rather than layoffs. Van Audenrode (1994) demonstrates that short-time compensation schemes must be sufficiently generous compared to mandatory severance payments and unemployment benefits in order for adjustment in hours only to be efficient. Thus, strict Employment Protection Legislation may make short-time work an efficient adjustment mechanism.

The effectiveness of short-time work will depend on their design and the context in which they are applied. To avoid deadweight loss (unwarranted hours subsidisation) and displacement effects (subsidisation of structurally inefficient matches), public authorities can adjust eligibility, compensation and duration. The effectiveness of short-time work also depends on other labour market institutions in the national setting. This explains why short-time work schemes differ widely across countries in terms of their generosity and eligibility and entitlement criteria (see Hijzen and Venn 2011).

To conclude, short-time work arrangements aim to limit inefficient separations during temporarily adverse demand conditions. Theory predicts that firms are more likely to enrol in such schemes if their employees are more skilled, have long tenure, are hired under permanent contracts and are protected by high dismissal costs. Short-time work institutions can vary substantially across jurisdictions in their design parameters and in their effectiveness. Often, the latter is determined by interactions with other policies such as EPL and bargaining institutions. All in all, however, publicly funded short-time work can be mutually beneficial for employers and employees, stabilising employment and income at the aggregate level while preserving otherwise viable firm-worker matches.

### Empirical evidence

Substantial research on short-time work and its effects was produced following the economic and financial crisis of 2008–2009. Labour adjustments to the recession differed across national settings, in particular the relative roles of reductions in employment (extensive margin) and in hours worked (intensive margin). The comparison between Germany and the U.S. captured the attention of many researchers. Although the size of the slump was comparable across the two countries, the employment response in Germany was considerably more muted, while the response of hours per worker was larger. However, Burda and Hunt (2011) and Möller (2010) downplay the contribution of short-time work to the muted response of employment in Germany. Both studies stress increased use of work time accounts.

Hijzen and Venn (2011) provide a cross-country study of the change in employment and average hours in relation to the take-up of short-time work during the recent recession. Of the 19 countries examined over 2003Q1-2009Q3, some had short-time work arrangements before the crisis, some adopted them and others did not. After controlling for the intensity of the recession, they estimate that countries with a short-time working scheme experienced a significantly more muted reduction in permanent employment. There is also evidence that average hours worked by permanent employees fell more in countries with short-time work arrangements. Hijzen and Venn (2011) show that the availability of short-time work arrangements cannot explain the different response of employment and average hours worked for temporary employees. However, the reduction in employment was considerably larger for temporary employees than for permanent employees in both sets of countries.

Boeri and Bruecker (2011) document the cross-country impact of short-time work schemes during the crisis (including Luxembourg). They report that short-time work take-up dampens the response of employment and identify a threshold at which short-time work begins to help prevent employment losses (GDP contraction of 1.5% or more). Using these parameter estimates, they calculate the number of jobs potentially saved by these schemes during 2008–2009. For Luxembourg, up to 0.3% of 2008 Q4 employment may have been saved. However, Boeri and Bruecker (2011) note that the application of the same coefficients across all countries may underestimate these effects for countries with efficient short-time work arrangements already in place.

Boeri and Bruecker (2011) also investigate how short-time work schemes interact with labour market institutions. They find that EPL strictness and bargaining centralisation indices have positive effects on national short-time work take-up rates, supporting the hypotheses presented in the previous section. They also investigate the impact of firm business conditions, structural characteristics, human capital investment and labour force composition on firm take-up rates. Using German establishment data for 2009, they find that both a fall in past sales and low expectations for future revenue negatively affect the take-up rate, while high competitive pressures induce firms to increase it. The authors conclude that short-time work “take-up rates are mainly affected by contemporaneous or anticipated shocks rather than by long-lasting structural problems”. Furthermore, the intensity of short-time work use increases with firm size, export share and share of research and development activities. Interestingly, the share of employees with higher educational attainment is associated with lower take-up, and the negative impact increases with the level of education. The share of employees on part-time or fixed-term contracts appears to reduce the share of firm employment in short-time work, supporting the hypothesis that permanent employees provide stronger incentives for firm participation. Finally, collective pay agreements do not have a clear effect on firm participation, although the impact may be negative.

Using the same dataset, Crimmann et al. (2010) turn to the question of firm selection into short-time work, or the extensive margin of participation. They find that, unlike in 2003, firms taking up short-time work in 2009 were less likely to have high shares of qualified or university-trained employees. They point to the specific nature of the 2008–2009 recession as a possible explanation, since it hit exporting manufacturing firms particularly hard and presumably these had large shares of non-specialised, blue-collar workers. Most of their other evidence also confirms Boeri and Bruecker’s (2011) findings on the intensive margin of take-up. These include a positive effect of establishment size and deteriorating performance (past profitability, future expectations) on the likelihood of using short-time work. In addition, flexible arrangements, such as part-time, fixed-term and agency contracts, tend to reduce the probability of participation. Exporting establishments are also more likely to participate in short-time work, even after controlling for other factors. Finally, they find no significant impact from the application of collective pay agreements.

## Short-time work in Luxembourg

### Institutional characteristics of the short-time work arrangements in Luxembourg

In Luxembourg, short-time work arrangements, also known as “partial unemployment” schemes, were introduced in the mid-1970 s following the onset of the steel crisis. There are different types of short-time work arrangements:

- Short-time work for economic reasons: applicable to firms that face a downturn in their activity, e.g. due to a temporary demand shock. The aim is to encourage labour hoarding and avoid layoffs.
- Short-time work due to economic dependence: applicable to firms whose activity strongly depends on one or more firms using short-time work.
- Short-time work for structural reasons: applicable to firms that face structural problems. The aim is to facilitate the adjustment process and limit layoffs.
- Short-time work due to “force majeure”: drop in production due to exceptional circumstances beyond the control of the firm (e.g. adverse weather conditions etc.).

While the following depiction of short-time work arrangements generally is true for all types of arrangements, we focus on the two short-time work arrangements related to economic fluctuations.

### General procedure

Firms apply for short-time work at the “*Comité de conjoncture*”, a tripartite committee including representatives of the government, employers’ organisations and trade unions.^3^ Firms are asked to provide the reason for their application, the expected duration and the number of employees (potentially) working short. Firms are also requested to provide detailed information on their economic and financial situation, e.g. their annual accounts. In case of short-time work for structural reasons, the application has to be accompanied by a restructuring plan. Firms must introduce a request for renewal every month. The committee’s secretariat collects the monthly applications, provides a preliminary assessment and evaluates the firm’s current economic, financial and social situation. This analysis enables also to assess the firm’s medium- to long-term prospects. At its monthly meeting, the tripartite committee evaluates all applications (for the upcoming month) individually and decides whether they are accepted or rejected.

Employees in short-time work are entitled to a compensatory allowance for lost working hours. The (monthly) wage for the hours worked and the compensation for the non-worked hours are paid by the employer. Short-time compensation amounts to 80% of the employees’ regular gross wages^4^ (up to a threshold of 250% of the statutory minimum wage). A firm whose application has been accepted by the tripartite committee may request reimbursement of the compensation for the hours not worked, except for the first 16 (8) h lost each month for full-time employees (part-time employees). Compensation for these initial hours lost is borne by the firm.

### Duration

Partial unemployment arrangements are limited in time. For short-time work due to economic reasons, the scheme cannot exceed 6 months within a 12-month reference period. For short-time work for economic dependency, the duration depends on the situation in the firm on which it depends. For short-time work due to structural problems, the duration of the scheme is defined within the firm’s restructuring plan. In any case, the individual reduction in working time is limited to 50% of the employees’ average working hours per month.

### Eligibility criteria

- Short-time work arrangements apply to firms of all size classes. In principle, public support is only available for those sectors that have been declared to be “in a crisis” by the government and on the basis of the tripartite committee’s proposal.^5^ Firms from other sectors may be eligible if they depend on firms in the short-time work scheme. Sectors that are considered as “highly competitive” are not eligible for short-time work.
- Short-time work arrangements are applicable to all permanent or fixed-term employees (including those working part-time). Agency workers and apprentices are excluded.

### Temporary changes during the crisis

Following the onset of the crisis in late 2008, the government decided to encourage employment retention and work sharing by temporarily modifying the existing short-time work schemes. These changes were originally intended to last until the end of 2009. However, as the recession continued to deepen, the government successively extended and scaled up the short-time work provisions over the years 2010–2015/2016:

- *Coverage was extended* e.g. to firms in sectors that have not been declared as being “in a crisis” (under certain conditions).
- *Duration was extended* the reference period was extended up to 12 months, i.e. the reduction in the working time was extended from 50% of the employees’ average monthly working hours to 50% of the employees’ average annual working hours (but capped at a maximum of 130 days per year).
- *Entitlements were enhanced* (for both employees and employers): compensation was extended to include the first 16 h lost^6^ and compensation could be increased for training during short-time work (see Table 6 in the Appendix 1 for more detailed information).

These changes certainly contributed to the increased use of short-time work after the crisis. However, due to the limited information available (from the survey or administrative data from the *Comité de conjoncture*), we cannot assess how many more applications occurred because of these legislative changes or whether the rise in applications would have occurred, even in a no-policy change scenario. Some firms apply for short-time work as a precautionary measure only, without necessarily resorting to the financial support. After approval, they may actually not implement short-time work. Also, firms may reduce the number of employees or the number of working hours lost relative to the request as approved. At the height of the crisis (in 2008–2009), administrative data suggest that 82% of those firms that had applied for short-time work (and whose applications were accepted), did effectively use it. In the subsequent period (2010–2013), this share slightly dropped to 79% of the applying firms.

### Performance of Luxembourg’s economy in 2008–2013

In the initial phase of the global economic and financial crisis, the Luxembourg economy plunged into a deep recession. In 2008–2009, real GDP fell by 8.2% peak to trough, a sharper drop than the euro area average (− 5.8%). This reflected Luxembourg’s exposure to financial services and the collapse in international trade (OECD 2010). After a short-lived rebound in 2010, real GDP slowed again in 2011 and receded the following year. Subsequently, Luxembourg’s economy has been growing rapidly at around 4% on average each year.^7^

While employment growth slowed down, it did not turn negative between 2008 and 2015 despite the sharp contraction in the export-oriented manufacturing sector, but also construction, transportation, as well as banking activity. In the second half of 2009 job creation effectively came to a standstill, with employment remaining virtually unchanged (excluding independent workers). Despite the severity of the GDP decline, employment adjustment remained small, reflecting significant labour hoarding. Firms’ preference to reduce hours worked rather than employment levels relates to extensive use of short-time work schemes (e.g. in manufacturing) and structural shortages of skilled labour (e.g. in the banking sector). In the latter case, firms’ reluctance to cut jobs may reflect expected difficulties in the recruitment of new employees with required skills in the next upturn. Cross-border workers, who account for more than 40% of total domestic employment, were particularly severely affected by the crisis. This is mainly related to the fact that cross-border workers are overrepresented in sectors with high shares of temporary contracts or internationally-oriented sectors (e.g. manufacturing, finance, business services and transportation).^8^

### Short-time work use during the economic and financial crisis

**Fig. 1:**
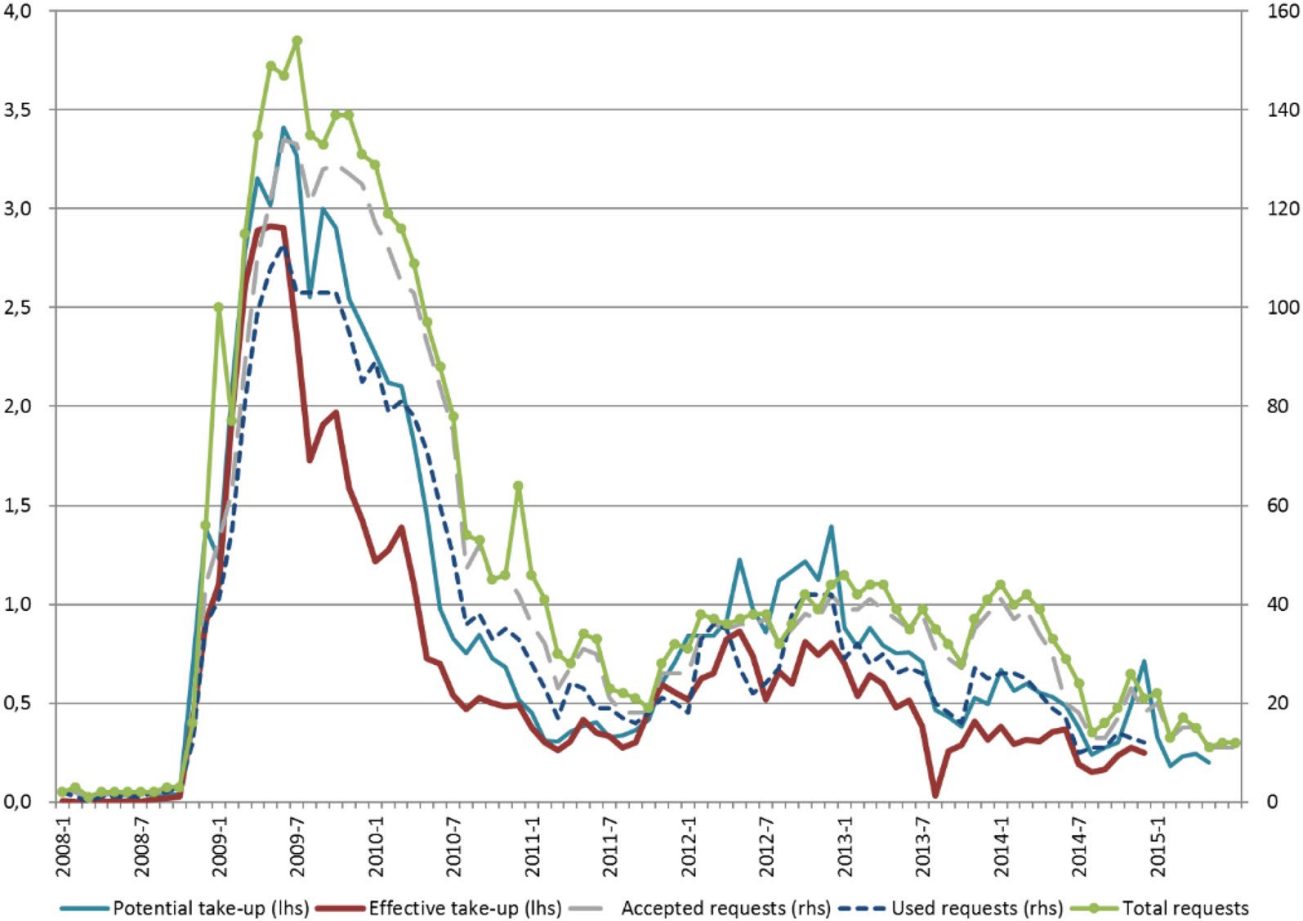
Participation in short-time work (left-hand scale: as a percentage of total employees, right-hand scale: absolute number of firms) (source: *Comité de conjoncture*, own calculations)

Administrative data shows that recourse to short-time work surged in the second half of 2008, in line with the sharp drop in economic activity (Fig. 1). At the height of the crisis (2008Q2–2009Q2), participation in short-time work peaked at nearly 4.5% of all employees^9^ (actual take-up rather than approval). Following a steady decline through 2011Q2, the use of short-time work rose again in the wake of the renewed weakness in demand resulting from the emergence of sovereign debt concerns in Europe (about 1% of employees participating).^10^ Despite the downward trend observed since 2013, short-time work schemes are still being requested by firms. The gap between potential and effective take-up may signal firm perceptions that the recovery continues to be fragile and that they may be using the scheme on a precautionary basis.

In 2009, short-time work participants lost on average nearly 30% of the usual working hours of a full-time worker (estimate based on monthly administrative data). The average reduction in hours worked per employee has been increasing (towards 40% in 2014), along with the gradual decline in the number of short-time work participants, probably reflecting diminishing work sharing over time.

Firms in the survey (details provided in the next section) were asked whether they applied for short-time work and whether their request was accepted. The share of firms applying for short-time work remained broadly stable in 2008–2009 and 2010–2013, at 1.5%–1.6%,^11^ but the proportion of requests that were accepted fell from 58% in 2008–2009 to 41% in the subsequent period (Table 1).

**Table 1:** Use of short-time work (percent of firms)

Sector	(Yes,) our firm applied [% of firms in the target population]		But the application was rejected [% of firms that applied]		And the application was accepted [% of firms that applied]	
	2008–2009	2010–2013	2008–2009	2010–2013	2008–2009	2010–2013
Total	1.6	1.5	42	59	58	41
Manufacturing	11.6	10.3	11	13	89	87
Construction	3.9	3.2	64	49	36	51
Trade	0.7	1.6	0	100	100	0
Business services	0.8	0.5	70	75	30	25

Weighted to be representative of the firm population. Financial intermediation is not shown in the table as all figures are nil

Q3.3b: Did your firm apply for short-time work since the beginning of 2008?

Applications for short-time work mostly originated in the manufacturing sector (where around 11% of firms applied) and were rarely refused.^12^ Rejection rates were much higher in construction, business services (and trade in 2010–2013). This reflects the legal provisions that govern the use of short-time work and how they were adapted as the crisis unfolded. Also, larger firms were more likely to apply and to be accepted.

## Data

This paper draws on a survey among Luxembourg firms that asked them about their labour input adjustment in response to the economic and financial crisis. The questionnaire (see Mathä et al. 2016) was directed at companies’ human resource managers and/or CEOs and collected firm characteristics as well as qualitative views on economic shocks and the firms’ use of STW. Most questions refer to two separate time periods; the years 2008–2009 cover the initial phase of the economic and financial crisis while the years 2010–2013 capture the European sovereign debt crisis.

The sample is derived from a target population of firms based on the Luxembourg firm register at the end of 2013. At the cost of possibly introducing a survival bias, the target population was restricted to firms in operation since end-2007.^13^ The target population was furthermore restricted to firms in the 5 sectors: manufacturing (NACE2: C), construction (NACE2: F), wholesale and retail trade (NACE2: G), business services (NACE2: H, I, J, L, M, N) and financial services (NACE2: K).^14^ The firms were categorised into the following size classes: “1–4 employees” (micro firms), “5–19 employees” (very small firms), “20–49 employees” (small firms), “50–199 employees” (medium-sized firms) and “200+ employees” (large firms). Some firms were directly included in the sample because they participated in similar surveys conducted in 2008 and 2009. The remaining firms in the sample were selected via a stratified random selection procedure, to ensure good coverage in all 25 strata (defined by the combination of sectors and size classes). The final sample collected contains 674 firms, representing a total response rate of 13.5%. The sample is post-stratified so that results are representative of either the target population of firms or the set of employees in the target firm population. In some cases, the size class provided by Luxembourg’s national statistics institute STATEC did not match those indicated by the firms. These firms were re-classified to the size class reported by the firm. However, the number of firms or employees in the target population was not adjusted.

The survey provides information on firms’ assessments of the impact of a set of external factors linked to the economic crisis on their activity, specifically the *level of demand*, *demand volatility*, *access to finance*, *customers’ ability to pay* and *availability of supplies*. Demand related factors were predominant in 2008–2009, during the initial phase of the crisis, when 36% of firms representing 33% of employment reported that their activity was negatively affected by demand (see Mathä et al. 2016, Table 4). In 2010–2013, *customers’ ability to pay* became the most relevant factor negatively affecting their activity (44% of firms), followed by demand-related shocks (41% of firms). Most Luxembourg firms were not (negatively) affected by the access to external finance and few firms reported a decrease in the availability of inputs from their usual suppliers.

The survey collects information on various structural characteristics of the firms to analyse how adjustments to the crisis vary across firm types. This information provides discriminating variables for the descriptive statistics reported below and covariates for regression analysis.

The survey suggests that Luxembourg firms mainly employ full-time workers with permanent contracts (88% in 2007 and 87% in 2013 in employment-weighted terms). Part-time workers with permanent contracts account for around 8% of employees (Table 2). The remaining 4% are employees with fixed-term contracts. Aggregate statistics do not indicate any striking changes in this composition. In 2007, 55% of employees were cross-border workers, i.e. employees living in the neighbouring countries and commuting across the border to work in Luxembourg (i.e. 45% were resident in Luxembourg). The share slightly increased to 57% in 2013 (all employment-weighted).^15^ Luxembourg is the EU country with the highest share of immigrants, so it is not surprising that only about one-fifth of employees are Luxembourg nationals. Firms reported that 56% of employees were highly skilled and that most were with the firm for more than 5 years (59%). The average age of the firms in the sample is almost 26 years.

**Table 2:** Labour force characteristics (percent of firms)

Share of type of employees in total	In 2007	In 2013	Skill level	
Permanent full-time	88	87	Higher skilled non-manual (ISCO: 1, 2, 3 and 7, 8)	56
Permanent part-time	8	9		
Temporary or fixed-term	4	4	**Job tenure**	
Total	100	100	Less than 1 year	11
Agency workers and others	45	43	Between 1 and 5 years	29
Cross-border workers	55	57	More than 5 years	59
Employees with Luxembourg nationality	23	22	Total	100

Data refer to the end of 2013 (unless otherwise stated). Aggregate statistics are weighted to be representative of the number of employees in the target firm population. Shares may not add up to 100% due to rounding

## Modelling firms’ decisions to apply for short-time work

In this section, we analyse the determinants of firms’ decision to apply for short-time work in Luxembourg.^16^ As we cannot distinguish between firms that would have been able to apply for STW under old and new STW rules, our analysis does generally not allow us to evaluate extended or new government policies; we analyse STW in general. We pool the 2008–2009 and 2010–2013 sub-periods into one regression, and estimate a logit specification as a function of external factors affecting firm activity (i.e. the shocks) as well as structural firm characteristics.^17^ The dependent variable is equal to 1 if the firm applied for short time work in period *t* (i.e. either 2008–2009 or 2010–2013) and 0 otherwise. We focus on negative shocks only since short-term work is in principle designed to aid firms facing a (temporary) fall in demand. We also include other negative shocks in the baseline specification without any strong a priori expectations about their effect.

Assume that the observed answer in the survey is related to the continuous latent variable y^*^ according to the following mapping:

$$y_{it} = \left\{ {\begin{array}{*{20}l} { 0 {\text{ if no STW application }}} \hfill \\ { 1 {\text{ if STW application}}} \hfill \\ \end{array} } \right.\begin{array}{*{20}c} {{\text{if }}y_{it}^{*} \le 0} \\ {{\text{if }}y_{it}^{*} > 0} \\ \end{array} ,$$

we estimate a logit model with

1 $${\text{Prob}}\left[ {y_{it} = 1} \right] = \frac{{\exp (x_{it} \beta + \varepsilon_{it} )}}{{1 + \exp (x_{it} \beta + \varepsilon_{it} )}} ,$$

where *ε*_*it*_ is the independently distributed error term. The set of covariates includes mainly variables related to the economic crisis and structural firm characteristics.

### Shock variables

The survey contains questions about five different shocks. The survey asked how firms’ activity in period *t* was affected by (i) the level of demand, (ii) the volatility of demand, (iii) the access to external finance, (iv) customers’ ability to pay and (v) the availability of supplies. The answer categories are formatted along a Likert scale ranging from 1 to 5, ranging from *strong decrease* (1), *moderate decrease* (2), *unchanged* (3), *moderate increase* (4) and *strong increase* (5). The use of STW is expected to be related to negative shocks; hence we create for each negative shock separately, various dummy variables assigning the value of 1 for firms experiencing (a) either a moderate or strong negative shock or (b) two separate dummy variables for (b1) moderate negative and (b2) strong negative shocks.^18^ Given the ordinal nature of the answers, various specifications were tried to assess the validity of various parameter restrictions, i.e. (not) assuming the probability to be linearly increasing in the ordinal scale, merging answers indicating increases/decreases or restricting attention to strong changes only. In the estimation table, we present specifications with various dummy specifications (Table 3).

**Table 3:**
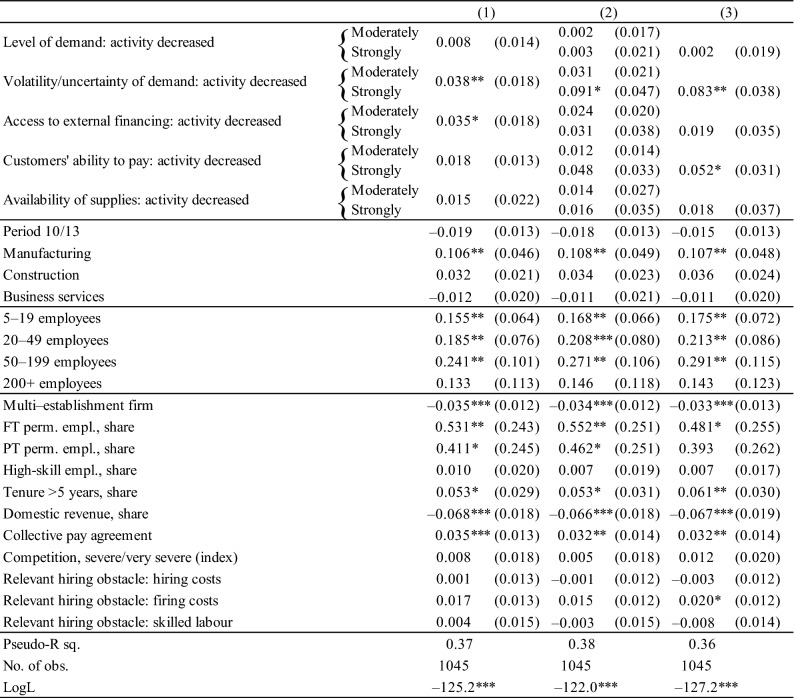
Pooled logit estimates for firms’ decision to apply for short-time work, marginal effects, 2008–2013

In column (1) moderate and strong negative shocks are pooled into a single dummy variable, while in column (2), they enter separately. Column (3) only considers strong negative shocks. Robust standard errors in (). The coefficients denote unweighted average marginal effects. ***,** and * indicate significance at the 1%, 5% and 10% level, respectively. Base category is trade, 1–4 employees, in 2008–2009, mainly foreign ownership, single-establishment

### Firm characteristics

*Firm size* is taken into account through the dummy variables indicating the size class. The base category is firms employing 1–4 employees, complemented by classes for 5–19, 20–49, 50–199 and 200+ employees. Since we expect that *collective pay agreements* might matter, we also include a dummy variable if a collective pay agreement of any kind (firm-level or outside the firm) was applied in 2013. Further, firm-specific variables include the *share of permanent full*-*time employees* and the *share of permanent part*-*time employees* in 2007. The share of fixed-term/temporary employees in that same year serves as base category. *Multi*-*establishment* firms can shift work and employees between plants, so we expect them to be less likely to be applying for short-time work. *Firing costs* for permanent employees, full-time or part-time, are expected to be higher than for temporary workers or agency workers. To maintain their firm-specific human-capital, firms with higher *shares of employees with at least 5 years of tenure *are expected to be more likely to apply for short-time work. Firms with more *skilled employees* and firms reporting *hiring costs* to be relevant obstacles to hiring new employees are also expected to be more likely to apply for short-time work.

## Estimation results

### Pooled estimates for both sub-periods

Firms facing strong declines in their activity due to demand volatility/uncertainty have a significantly higher likelihood of applying for short-time work (Table 3). This is the only external shock consistently associated with application for short-time work in Luxembourg. Taken at face value, this result seems to suggest that STW is primarily requested and used by firms facing demand fluctuations or uncertainty rather than a demand level shock, and thus STW is used as intended by the public authorities without impeding structural adjustments to lasting negative demand shocks. After controlling for other factors, firms in manufacturing are more likely to apply for short-time work, consistent with our descriptive findings and the fact that short-time work was initially only applicable in this sector. Larger firms are also more likely to apply for short-time work.^19^ Similar to Boeri and Bruecker (2011) and Crimmann et al. (2010) who used German establishment data, the probability of Luxembourg firms to apply for short-time work increases with the share of revenue originating in exports. However, we fail to find a significant impact of the share of skilled workers on the likelihood of applying for short-time work. The share of employees with at least 5 years’ tenure, which may also proxy for firm-specific human capital, does have a positive effect. Moreover, we find weak support for the notion that the relevance of firing costs as an obstacle to permanent hiring increases the probability of applying for short-time work (however, the effect is significant only in one of our specifications).

Furthermore, we find that the probability of applying for short-time work significantly increases with the shares of both full-time and part-time permanent contracts vis-à-vis fixed-term contracts (the reference group in the regression), a result also reported for German establishments by Boeri and Bruecker (2011) and Crimmann et al. (2010). However, unlike these two studies, we also find that in Luxembourg, firms are more likely to apply for STW if they are bound by collective pay agreements. Taken together, we interpret this as a sign that firms with less flexible contractual arrangements are more constrained in carrying out adjustments through the extensive margin and therefore more likely to apply for short-time work. This is also consistent with the probability of applying for short-time work being lower among multi-establishment firms or firms with a lower share of permanent employees, indicators that can be interpreted as mirroring the internal flexibility of such firms.

### Robustness

We modify the definition of the dependent variable in our pooled logit model and only consider firms whose application for STW was accepted (rather than all firms that applied). We also note that all firms in our sample that were accepted by the tripartite *Comité de conjoncture* also made use of STW. There is thus no evidence in our sample that firms would apply for STW for precautionary reasons, i.e. firms applying for STW and despite being accepted not making use of it.

The results presented in Table 9 in Annex 3 are broadly in line with our findings for all firms that applied for STW. In terms of shocks, the results are robust to the change in the definition of the dependent variable; firms facing strong negative impact on their activity due to both demand volatility and customers’ ability to pay are significantly more likely to use STW. Nevertheless, there are few differences. The effect of the largest size class on STW use is significant and in line with the estimates for the 2008–2009 period (Table 10). Firms with higher shares of high-skilled employees are significantly less likely to use STW. In the estimation on STW applications, the marginal effect was insignificant. The negative effect is in line with findings for Germany. Boeri and Bruecker (2011) report a negative effect of education levels on STW take-up, Crimmann et al. (2010) do so for qualified or university-trained employees. The latter in particular argue that this may be due to the specific nature of the 2008–2009 recession, hitting export-oriented manufacturing firms particularly hard, which presumably had larger shares of non-specialised blue-collar workers.

So far, we assumed that the determinants of firms’ decisions to apply for short-time work are the same in both periods. To assess whether this is the case, we estimate our model separately for the two sub-periods. Unfortunately, unlike the information on the shocks, the survey does not provide information on firm characteristics for separate sub-periods (see Table 8). In Tables 10 and 11 in Appendix 3, we assess whether firms that applied for short-time work experienced different shocks in the two sub-periods. In the 2010–2013 period (Table 11), firms facing a decline in their activity due to demand volatility/uncertainty or strong deterioration in access to external finance were significantly more likely to apply for short-time work. These results are in line with the pooled estimates in Table 3. In contrast, in the 2008–2009 period (Table 10), firms facing a deterioration in customers’ ability to pay and access to external finance were more likely to apply for short-timework. There is also a weak evidence that firms facing negative demand level and volatility shock were more likely to apply for STW, however, the result is not robust across different specifications. These separate regressions also elicit why the largest firms (i.e. firms with 200+ employees) had no significant impact on the likelihood of applying for short-time work in the pooled model (Table 3). In the 2010–2013 sub-period, not one single firm in this size class in our sample applied for STW. In the 2008–2009 sub-period (Table 10), the coefficient on the 200+ size category is significant and of a similar magnitude as the remaining size classes.^20^

Finally, we consider clustering standard errors at the firm level, as the error term of a firm in the first period may be correlated with its error term in the second period. The results are robust with the exception of the coefficient estimates of the share of permanent part-time employees and the indicator for long tenure. Both have slightly larger standard errors and turn insignificant at the conventional confidence levels. At the same time, the negative coefficient for the period 2010–13 becomes significant, which is in line with the descriptive evidence in Fig. 1.

## Effects of short-time work on employment and jobs saved

The increased use of short-time work during the recent crisis may have helped to preserve jobs. Estimating this impact, however, is challenging given the data available as the necessary counterfactual scenario is not easy to model. Therefore, we included a specific question in the survey to collect human resource managers’ and/or CEOs’ direct assessment of the effectiveness of short-time work in preventing job losses in their firms. They were asked to provide the number of employees involved in this scheme and the number of employees that would have been laid off had it been unavailable. In 2008–2009, firms reported that 25% of employees involved in short-time work would have lost their job without this arrangement. In 2010–2013, this share was still reported to be at 20%.

Administrative data from the *Comité de conjoncture* in Table 4 show that at its peak, 9630 and 4644 employees were effectively involved in STW in Luxembourg in 2008–2009 and 2010–2013, respectively. Using the self-reported shares of jobs saved, this would imply STW in Luxembourg to have saved 2400 and 921 jobs, respectively, corresponding to 0.7% and 0.3% of employment in the peak months of the respective sub-period.^21^

**Table 4:** Use of STW and its costs

Period	Number of employees effectively affected^a^	Working hours effectively lost	Share of affected jobs saved^b^	Estimated jobs saved^b^		Direct annual budgetary costs		
				Number	Pct. of employment	Cost (EUR million)	per job saved (EUR)	per hour lost (EUR)
2008	385		24.9	96	0.03	2.65	27,633	
2009	6998	3,881,860	24.9	1744	0.53	61.51	35,268	15.8
2010	2641	1,380,336	19.8	524	0.16	22.48	42,926	16.3
2011	1313	869,969	19.8	260	0.07	15.60	59,932	17.9
2012	2478	1,585,717	19.8	491	0.14	28.71	58,415	18.1
2013	1623	1,168,862	19.8	322	0.09	21.29	66,127	18.2
Peak use in the two subperiods								
May 2009	9630	441,002	24.9	2400	0.73			
March 2010	4644	201,809	19.8	921	0.28			

*Comité de conjuncture* for the number of employees affected and hours lost and annual activity reports of the Ministry of Labour for the budgetary costs

^a^Annual average of monthly number of employees affected

^b^Survey data or survey-based estimates

In practice, the share of saved employees might vary across sectors, size classes etc. While the data from the *Comité de conjuncture* is not available at a more disaggregated level, we can use the survey data for a more granular assessment of the possible impact of short-time work on employment. In doing so, we assume that the proportion of jobs saved in each stratum (defined by sector and size class combinations) is the same as in the employment in the entire population of the stratum. Clearly, this assumption might be too strong for strata with few firms in the sample or a low response rate. Indeed, Table 5 suggests that only in one stratum (manufacturing firms with 50–199 employees) we might have enough responses to make reliable inferences about the impact of short-time work in the population (out of 72 firms in the population in this stratum in 2010–2013, 16 replied to the questionnaire, of which 7 used short-time work). In fact, in this stratum short-time work has a measurable impact on employment: firms in the sample reported that STW saved 4.3% of their total employment in 2008–2009 (4.9% in 2010–2013). Extrapolating to the population of this stratum, this would correspond to about 318 jobs in 2008–2009 and 362 jobs in 2010–2013.

**Table 5:** Use of STW by stratum

Sector	Size class	Population		Sample							Extrapol. to population
				All sample		STW firms		STW employees			
		N	L	n	l	n^STW^	l^STW^	Involved	Saved	Saved [% of l]	Saved in L
2008–2009											
Manufacturing	5–19	268	2675	26	243	2	13	15	4	1.6	44
Manufacturing	20–49	99	3079	10	343	2	36	27	5	1.5	45
Manufacturing	50–199	82	7920	16	1743	4	523	305	70	4.0	318
Manufacturing	200+	32	23,399	4	1159	2	631	180	50	4.3	1009
Construction	5–19	819	8336	47	547	1	5	6	4	0.7	61
Construction	20–49	306	9276	59	1829	2	69	35	6	0.3	30
Trade	5–19	1257	11,246	53	562	1	8	4	2	0.4	40
Trade	20–49	226	7008	42	1314	1	38	3	3	0.2	16
Trade	50–199	76	5500	14	1228	1	68	50	10	0.8	45
Business services	20–49	195	5759	41	1398	2	51	45	13	0.9	54
2010–2013											
Manufacturing	5–19	193	1968	26	243	2	38	26	5	2.1	40
Manufacturing	20–49	91	2883	10	343	1	43	20	5	1.5	42
Manufacturing	50–199	72	7332	16	1743	7	807	433	86	4.9	362
Construction	5–19	705	7092	47	547	1	14	7	7	1.3	91
Construction	20–49	287	8854	59	1829	2	80	24	9	0.5	44
Construction	50–199	121	10,612	20	1587	1	72	65	1	0.1	7
Business services	20–49	419	12,865	41	1398	1	20	20	5	0.4	46

N and L denote the total no. of firms and employees in the target firm population; n and l denote the total no. of firms and employees in the sample; n^STW^ and l^STW^ denote the number of firms and employees with short-time work. The data for the firm population refers to end 2008 and end 2013. Thus, the number of employees in short-time work can exceed the number of total employees in a firm. Size categories based on 2013 employment figures

If we consider all size classes in manufacturing where at least one respondent firm used short-time work (see Table 5), this arrangement saved 3.8% of employment in the population in 2008–2009 and 3.6% in 2010–2013. At the peak of short-time work take-up (2008–2009), the estimated number of jobs saved in manufacturing is 1416. The number falls to 444 in 2010–2013 because we have no response from manufacturing firms with 200+ employees.

To the best of our knowledge, the only other estimates of jobs saved through STW in Luxembourg are derived from macroeconomic data. Boeri and Bruecker (2011) apply the estimated coefficients from a cross-country regression and calculate the number of the jobs saved by STW during the Great Recession in Luxembourg at 0.3% of employment or 552 employees. They also note that this approach may underestimate the true effects in countries with efficient STWAs in place. Indeed, our results derived from the self-reported figures by firms suggest much higher number of jobs saved in 2008–2009 (about 1400 in Manufacturing and 2400 in the whole economy).

### Costs and benefits of STW in Luxembourg

Next, we consider the costs and benefits of STW in Luxembourg. The costs include the direct budgetary costs of short-time work (covering compensation for the hours not worked in the STW framework) and administrative costs of running the programme. Publicly available data cover only the first type of costs. The direct benefits of STW are related to the unemployment benefits that would have been payed to employees losing their job otherwise. Other benefits of STW include retained firm-specific human capital of the employees that would have been fired, a general loss of human capital during longer spells of unemployment, discouraged workers etc. We abstract from both administrative costs and indirect benefits, as such data are unavailable, and provide a first tentative and partial assessment.

According to Table 4, the direct budgetary costs of short-time work in Luxembourg rose from €16 per hour lost in 2009 to €18.2 in 2013. This implies that the average cost of one job saved increased from approximately €2900 per month in 2009 to €5500 in 2013. From a government perspective, the direct benefit of STW consists of lower payments of unemployment benefits to those who would have lost their job otherwise. As discussed earlier, the situation of Luxembourg is specific because about half of the labour force are cross-border workers. If they lose their employment in Luxembourg, their unemployment benefits are paid by their respective country of residence. At the same time, during the Great Recession cross-border workers were more severely affected by job losses. This impedes us from precisely calculating the budgetary costs of paying unemployment benefits to people having been laid off in the absence of STW availability. As an approximation and an upper bound, we assume that one half of the employees that would have lost their job in the absence of STWA would have received unemployment benefits in Luxembourg (i.e. we consider the share of cross-border workers in the private sector employment in Luxembourg and assume that resident and cross-border workers have the same likelihood of being involved in STW and losing their job in its absence). Focusing on the peak period of STW use in 2009, this would imply approximately additional 870 unemployed and annual budgetary cost of €27.6 million. Hence, from a pure Luxembourg perspective, the rough approximation of benefits of the STW programme lie below its direct costs. If we include in the approximation the budgetary costs of the cross-border workers saved through STW in Luxembourg, we would arrive at a figure that is closer but still below the direct costs of the STW programme. The benefit of STW in terms or lower budget costs related to otherwise higher unemployment only applies as long as the people remain unemployed. A more sophisticated cost–benefit analysis would have to take into account not only the average duration of unemployment spells but also consider social costs of unemployment such as the deterioration of human capital during unemployment, long-term unemployment etc.

## Concluding remarks

The economic and financial crisis led to an unprecedented surge in the number of Luxembourg firms using short-time work. Firms reported that 25% of employees involved in short-time work would have lost their job without this arrangement in 2008–2009 and 20% would have lost it in 2010–2013. Economy-wide, this would translate to approximately 2400 and 920 jobs saved in each sub-period, respectively. This corresponds to 0.7% and 0.3% of employment in the respective sub-periods.

Short-time work in Luxembourg is concentrated in the manufacturing sector, in which 1.5% to 4.9% of total jobs were saved through short-time work, depending on the size class and period. The likelihood that a Luxembourg firm applied for or used short-time work is higher for single establishment firms and firms reporting negative impact of demand volatility/uncertainty and customers’ ability to pay on their activity. In addition, the likelihood of the use of short-time work increases with the share of permanent employees and the degree of export orientation. Taken at face value, the results suggest that STW is primarily requested and used by firms facing demand fluctuations or uncertainty rather than a demand level shock. Furthermore, STW is used by firms with high levels of firm-specific human capital and firms constrained by collective pay agreements, and thereby helps to avoid costly and inefficient separations of employees. Altogether, this suggests that, in Luxembourg, STW is used as intended; we do not find evidence pointing towards STW use impeding structural adjustments to lasting negative demand shocks.

## Figures and Tables

**Table 9:**
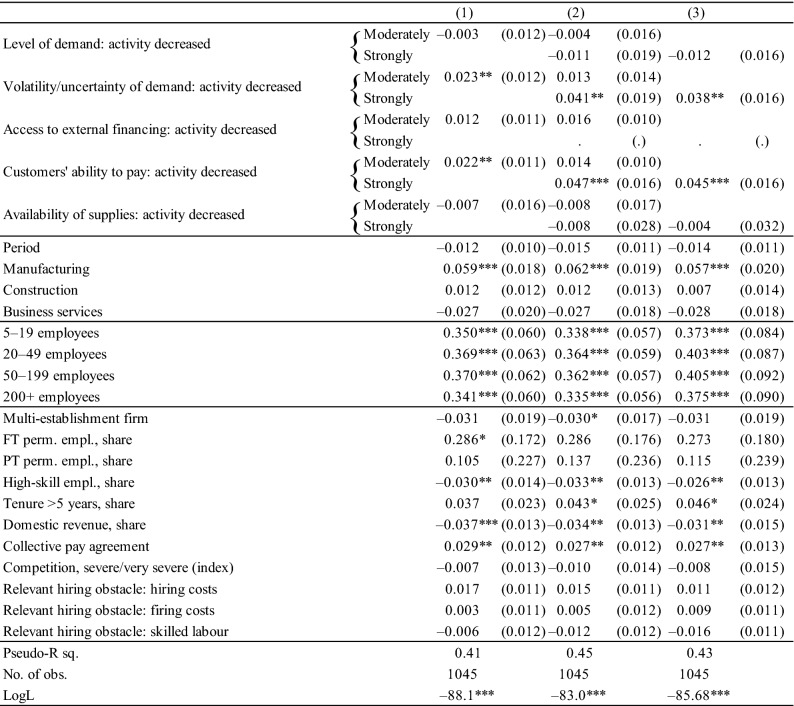
Logit estimates for firms whose application for short-time work was accepted (and used), marginal effects

See Table 3. Strong negative access to finance shock is omitted because it completely determines the outcome

**Table 10:**
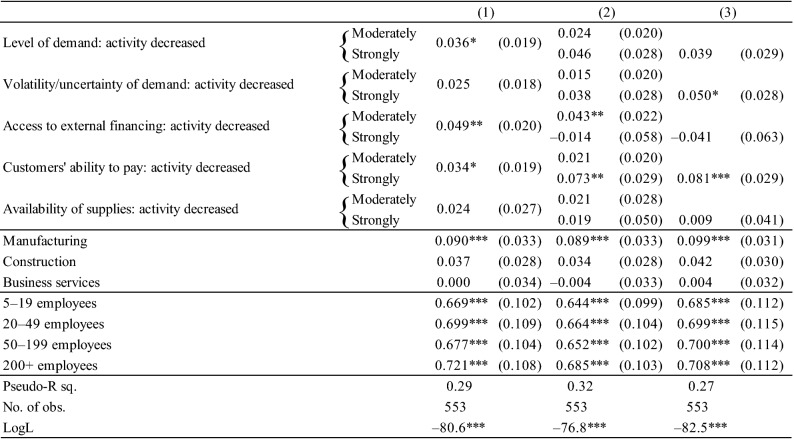
Logit estimates for firms’ decision to apply for short-time work in 2008–2009, marginal effects

See Table 3

**Table 11:**
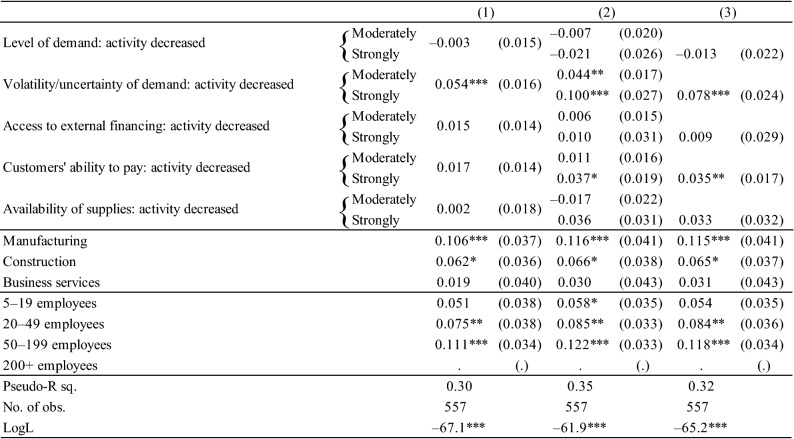
Logit estimates for firms’ decision to apply for short-time work in 2010–2013, marginal effects

See Table 3. The coefficient for the size class 200+ employees was not estimated as no firm in this size class in our sample applied for STWA in the 2010–2013 period

## Appendix 1: Characteristics of short-time work arrangements in Luxembourg

See Table 6.

**Table 6:** Changes applied to the existing short-time work schemes

Period of application (effective date)	Eligibility requirements (firms)	Duration	Compensation
Economic reasons/economic dependency			
Jan. 2009–Dec. 2009 (Mar. 2009)^a^	–	The reduction in the employees’ average working time may exceed 50% per month, provided that it does not exceed 50% of the employees’ annual average working time (with a maximum duration of 6 months within a 12-month reference period)	Compensation was extended to the first 16 h lost per month (for full-time employees, to the first 8 h lost per month for part-time workers)
Jan. 2009–Dec. 2010 (May 2009)^b^	–	The reduction in the employees’ average working time may exceed 50% per month, provided that it does not exceed 50% of the employees’ average annual working time	Idem
Aug. 2010–Jul. 2012 (Aug. 2010)^c^	Eligibility was extended to firms which do not belong to eligible sectors but which face a reduction in their working time of at least 40% and provided that they concluded an *Employment maintenance plan* or upon agreement with the social partners (applicable from August 2010 to July 2012)	The reduction in the employees’ average working time may exceed 50% per month, provided that it does not exceed 50% of the employees’ average annual working time (prolonged until December 2011)	Compensation was extended to the first 16 h lost per month (for full-time employees, to the first 8 h lost per month for part-time workers) (prolonged until December 2011) Employers’ social security contributions are reimbursed for firms that have been on short-time work for the last 6 months and that face a reduction in their normal working time of at least 25% (per month) (applicable from August 2010 to July 2012)
Jan. 2012–Dec. 2012 (Dec. 2011)^d^	–	Idem (prolonged until December 2012)	Compensation was extended to the first 16 h lost per month (for full-time employees, to the first 8 h lost per month for part-time workers) (prolonged until December 2012)
Aug. 2012–Dec. 2013 (Jul. 2012)^e^	Idem (prolonged until December 2013)	Idem (prolonged until December 2013)	Compensation was extended to the first 16 h lost per month (for full-time employees, to the first 8 h lost per month for part-time workers) (applicable from August 2012 to December 2013) Employers’ social security contributions are reimbursed for firms that have been on short-time work for the last 6 months and that face a reduction in their normal working time of at least 25% (per month) (prolonged until December 2013)
Jan. 2014–Dec. 2015 (Dec. 2013)^f^	Idem (prolonged until December 2015)	Idem (prolonged until December 2015)	Compensation was extended to the first 16 h lost per month (for full-time employees, to the first 8 h lost per month for part-time workers) (applicable from August 2012 to December 2015) Employers’ social security contributions are reimbursed for firms that have been on short-time work for the last 6 months and that face a reduction in their normal working time of at least 25% (per month) (prolonged until December 2015)
Structural reasons			
Jan. 2009–Dec. 2009 (Mar. 2009)^a^	–	–	Compensation was extended to the first 16 h lost per month (for full-time employees, to the first 8 h lost per month for part-time workers), provided that the firm concluded an *Employment maintenance plan* or upon agreement with the social partners
Jan. 2009–Dec. 2010 (May 2009)^b^	–	–	Idem
Aug. 2010–Jul. 2012 (Aug. 2010)^c^	–	–	Compensation was extended to the first 16 h lost per month (for full-time employees, to the first 8 h lost per month for part-time workers), provided that the firm concluded an *Employment maintenance plan* or upon agreement with the social partners (prolonged until December 2011)
Jan. 2012–Dec. 2012 (Dec. 2011)^d^		Idem (prolonged until December 2012)	Idem (prolonged until December 2012)
Jan. 2013–Dec. 2013 (Jul. 2012)^e^		The reduction in the employees’ average working time may exceed 50% per month, (i) provided that it does not exceed a duration of 10 months over a reference period of 12 months and (ii) provided that a restructuring plan is added to the *Employment maintenance plan*	Idem (prolonged until December 2013)
Jan. 2014–Dec. 2014/2015 (Dec. 2013)^f^	–	Idem (prolonged until December 2014)	Idem (prolonged until December 2015)
Jan. 2015–Dec. 2016 (Dec. 2014)^g, h^	–	Idem (prolonged until December 2016)	Idem (prolonged until December 2016)

References: ^a^Legilux. (2015a). ^b^Legilux. (2015b). ^c^Legilux. (2015c). ^d^Legilux. (2015d). ^e^Legilux. (2015e). ^f^Legilux. (2015f). ^g^Legilux. (2015g). ^h^Legilux. (2015h)

## Appendix 2: Variables definition and summary statistics

See Tables 7 and 8.

**Table 7:** Variables definition and summary statistics (economic shocks)

	N	Mean
Description: 1 if factor firms’ activity … in t, 0 otherwise		
Level of demand		
Decreased	1045	0.34
Moderately	1045	0.22
Strongly	1045	0.13
Volatility/uncertainty of demand		
Decreased	1045	0.29
Moderately	1045	0.21
Strongly	1045	0.09
Access to external financing		
Decreased	1045	0.19
Moderately	1045	0.14
Strongly	1045	0.05
Customers’ ability to pay		
Decreased	1045	0.35
Moderately	1045	0.27
Strongly	1045	0.08
Availability of supplies		
Decreased	1045	0.11
Moderately	1045	0.09
Strongly	1045	0.02

**Table 8:** Variables definition and summary statistics

Variable	Description	N	Mean
Applied for STW	Discrete; 1 if applied for STW in t, 0 otherwise	1045	0.05
Period 10/13	Discrete; 1 if t is 2010/13, 0 otherwise	1045	0.50
Manufacturing	Discrete; 1 if firm belongs to NACE code C, 0 otherwise	1045	0.13
Construction	Discrete; 1 if firm belongs to NACE code F, 0 otherwise	1045	0.25
Trade	Discrete; 1 if firm belongs to NACE code G, 0 otherwise	1045	0.26
Business services	Discrete; 1 if firm belongs to NACE codes H, I, J, L, M or N, 0 otherwise	1045	0.35
1–4 employees	Discrete; 1 if firm had 1–4 employees at the end of 2013, 0 otherwise	1045	0.20
5–19 employees	Discrete; 1 if firm had 5–19 employees at the end of 2013, 0 otherwise	1045	0.34
20–49 employees	Discrete; 1 if firm had 20–49 employees at the end of 2013, 0 otherwise	1045	0.27
50–199 employees	Discrete; 1 if firm had 50–199 employees at the end of 2013, 0 otherwise	1045	0.14
200+ employees	Discrete; 1 if firm had 200 employees or more at the end of 2013, 0 otherwise	1045	0.04
Age of firm	Continuos, in years derived from the first year of operation	1045	25.70
Multi-establishment firm	Discrete; 1 if the firm was a multiple-establishment firm at the end of 2013, 0 otherwise	1045	0.12
FT perm. empl., share	Continuous; permanent full-time employees as a share of total employees at the end of 2007	1045	0.88
PT perm. empl., share	Continuous; permanent part-time employees as a share of total employees at the end of 2007	1045	0.10
High-skill empl., share	Continuous; employees belonging to ISCO classes 1, 2, 3, 7 or 8 as a share of total employees at the end of 2013	1045	0.60
Tenure > 5 years, share	Continuous; employees with job tenure exceeding 5 years as a share of total employees at the end of 2013	1045	0.61
Domestic revenue, share	Continuous; sales in the domestic market as a share of total sales in 2013	1045	0.75
Collective pay agreement (based on 4.3)	Discrete; 1 if the proportion of employees covered in 2013 by any collective pay agreement is greater than 0, 0 otherwise	1045	0.39
Competition, severe/very			
severe (index)	Continuous, weighted average; 1 = weak, 2 = moderate, 3 = severe, 4 = very severe, weighted by the respective market share in 2013	1045	0.80
Relevant hiring obstacle: hiring costs	Discrete; 1 if firing costs were a relevant/very relevant obstacle in hiring workers with a permanent, open-ended contracts at the end of 2013, 0 otherwise	1045	0.35
Relevant hiring obstacle: firing costs	Discrete; 1 if hiring costs were a relevant/very relevant obstacle in hiring workers with a permanent, open-ended contracts at the end of 2013, 0 otherwise	1045	0.50
Relevant hiring obstacle: skilled labour	Discrete; 1 if insufficient availability of labour with the required skills was a relevant/very relevant obstacle in hiring workers with a permanent, open-ended contracts at the end of 2013, 0 otherwise	1045	0.67

## Appendix 3: Additional results

See Tables 9, 10 and 11.

## Footnotes

1 The evolution, determinants and effects of other labour market policy measures in Luxembourg during the crisis are analysed separately in Efstathiou et al. (2018). In principle, other labour market policies do not influence applications to short-time work as these programmes have different purposes. The role of short-time work schemes is to preserve existing jobs while active labour market policies focus on promoting job creation.

2 Boeri and Bruecker (2011) estimate jobs saved by STWA in Luxembourg using macroeconomic data.

3 See Legilux (2015a, b, c, d, e, f, g, h).

4 Compensation is increased to 90% of the regular gross wage if the employee accepts to participate in vocational training.

5 Even in these cases, firms have to apply every month to continue benefiting from public support.

6 This applies to short-time work for economic reasons, economic dependency or structural problems; the latter however only if the firm agrees to an E*mployment maintenance plan*.

7 Over the period 2010–2013 (2014–2015), real GDP rose by 0.7% (1.8%) on average each year in the euro area. In the four largest euro area economies, GDP average growth rate amounted to 2.2% (2.0%) in Germany and 1.3% (1.1%) in France. Spain and Italy were still in a recession in 2010–2013 (with real GDP declining by 1.4% and 0.6% respectively, on average each year over this period). Economic growth turned however positive again in the 2 following years, with real GDP growing by 2.5% and 0.5% on average each year in Spain and in Italy respectively (Source: Eurostat Annual national accounts database).

8 See for example BCL (2012) for a detailed analysis of the impact of the crisis on Luxembourg’s labour market.

9 Total employees excluding agriculture and NACE2 sectors O–U.

10 Before the crisis, the use of short-time work was negligible (and concerned almost exclusively manufacturing firms). Between 2005 and the first half of 2008, there were only four applications for short-time work, on average each month (among which, three were, on average, effectively used). As regards the number of employees effectively working short, it increased from 25 (on average each month) in the first half of 2008 to nearly 800 in the second half of that year and 7000 in 2009.

11 To be precise, we assume that the proportion of firms with STW in each stratum in the sample is identical to the proportion in the population of the stratum. Estimates based on data from the *Comité de conjoncture* are more or less in line with these figures, suggesting that around 1.4% of firms applied for short-time work in the period 2008–2009, on average, each month (i.e. 65 firms on average in absolute terms). For 2010–2013 however, administrative data indicate a lower share (less than 1%, i.e. 47 firms). The discrepancy is likely to come from weighting of the survey answers. At the height of the crisis, in mid-2009, this number amounted to nearly 150 firms (see Fig. 1).

12 This is also confirmed by available data from the *Comité de conjuncture*: at the height of the crisis, in 2008–2009, average acceptance rates amounted to 99% in manufacturing and 70% in the remaining sectors of the economy. See also STATEC (2009).

13 Information for firms that discontinued their operations after 2007 could not be obtained. Firms that started their operation after 2007 could in principle have been included in the sampling population. This would however have unduly complicated the weighting. In addition, changes in survey results between the two periods would need to be decomposed into true changes in firm behaviour and compositional changes.

14 Non-market sectors (in particular public services) are excluded from the survey as they are not in the target sampling frame. The survey was aimed at firms in the private, non-farm sectors, excluding NACE codes above N. Financial services firms are included in the sample. However, none of the responding firms applied to or made use of short-time work. In the empirical estimations, financial services firms are excluded, as we include sector controls.

15 These figures are broadly in line with administrative data (reporting around 53% of cross-border workers). Source: Authors’ calculations based on social security data (IGSS).

16 Results were similar when the dependent variable was limited to applications that were approved.

17 We also experimented with including firm-specific random effects, but felt that this puts too much strain onto the firm-specific variables. In other words, the firm-specific random effects absorb too much variability given that we only have two periods at our disposal.

18 For a summary of these variables, see Table 7 in Appendix 2.

19 The coefficient for firms with 200+ employees is significant in the 2008–2009 sub-period but cannot be estimated for the period 2010–2013 because no firm in this category in our sample applied for STW in 2010–2013. For more details, see Sect. 6.2.

20 In 2008–2009, only two companies in the 200+ employee category in our sample applied for STWA. Hence, the results for this category should be interpreted with caution.

21 Using the number of jobs saved through STWA in Luxembourg, one could estimate the impact of short-time work on unemployment. However, this is challenging given that cross-border workers represent around 45% of total domestic employment in Luxembourg. Short-time working schemes are not restricted to resident workers and cross-border workers may equally benefit from it. At the same time, when the latter lose their job in Luxembourg, they are regarded and counted as unemployed in their respective country of residence. Hence, the number of cross-border jobs saved due to short-time work has an impact on domestic employment figures but no impact on the unemployment rate in Luxembourg.

## References

[CR1] ADEM (2015). Agence pour le développement de l’emploi. Rapport annuel.

[CR2] Arpaia A, Curci N, Meyermans E, Peschner J, Pierini F (2010). Short time working arrangements as response to cyclical fluctuation.

[CR3] BCL (2012). Le marché du travail luxembourgeois et la crise, BCL Bulletin 3. Encadré.

[CR4] Boeri T, Bruecker H (2011). Short-time work benefits revisited: some lessons from the Great Recession. Econ. Policy.

[CR5] Burda MC, Hunt J (2011). What explains the German labor market miracle in the Great Recession?. Brook. Pap. Econ. Act..

[CR6] Burdett K, Wright R (1989). Unemployment insurance and short-time compensation: the effects of layoffs, hours per worker, and wages. J. Pol. Econ..

[CR7] Crimmann, A., F., Wießner, F., Bellmann, L.: The German work-sharing scheme: an instrument for the crisis. ILO conditions of work and employment programme series 25 (2010)

[CR8] Efstathiou K, Mathä TY, Veiga C, Wintr L (2018). The use of active labour market policies: evidence from a survey among Luxembourg firms.

[CR9] Hall RE, Lazear EP (1984). The excess sensitivity of layoffs and quits to demand. J. Labor Econ..

[CR10] Hijzen A, Venn D (2011). The role of short-time work schemes during the 2008–09 recession, OECD social, employment and migration working papers.

[CR12] Legilux. *Loi du 17 février 2009*. Mémorial. Journal Officiel du Grand-Duché de Luxembourg, recueil de législation. Mém. n°35 du 02 mars 2009, p.444 (2015a)

[CR13] Legilux. *Loi du 29 mai 2009*. Mémorial. Journal Officiel du Grand-Duché de Luxembourg, recueil de législation. Mém. n°122 du 4 juin 2009, p.1736 (2015b)

[CR14] Legilux. *Loi du 3 août 2010*. Mémorial. Journal Officiel du Grand-Duché de Luxembourg, recueil de législation. Mém. N° 137 du 13 août 2010, p. 2212 (2015c)

[CR15] Legilux. *Loi du 16 décembre 2011*. Mémorial. Journal Officiel du Grand-Duché de Luxembourg, recueil de législation. Mém. n° 260 du 20 décembre 2011, p. 4324 (2015d)

[CR16] Legilux. *Loi du 31 juillet 2012*. Mémorial. Journal Officiel du Grand-Duché de Luxembourg, recueil de législation. Mém. n° 169 du 14 août 2012, p. 2584 (2015e)

[CR17] Legilux. *Loi du 23 décembre 2013*. Mémorial. Journal Officiel du Grand-Duché de Luxembourg, recueil de législation. Mém. n° 227 du 27 décembre 2013, p. 4241 (2015f)

[CR18] Legilux.*Loi du 19 décembre 2014*. Mémorial. Journal Officiel du Grand-Duché de Luxembourg, recueil de législation. Mém. n° 246 du 23 décembre 2014, p. 4804 (2015g)

[CR19] Legilux. *Loi du 24 décembre 2015*. Mémorial. Journal Officiel du Grand-Duché de Luxembourg, recueil de législation. Mém. n° 254 du 23 décembre 2014, p. 6174 (2015h)

[CR20] Mathä, T.Y., Veiga, C., Wintr, L.: Employment, wages and prices: How did firms adjust during the economic and financial crisis: evidence from a survey of Luxembourg firms. BCL Working Paper 104, Central Bank of Luxembourg (2016)

[CR21] Ministère du travail, de l’Emploi et de l’Economie sociale et solidaire. various years. Rapports d’activité 2008–2017. https://mteess.gouvernement.lu/fr/publications.html

[CR22] Möller J (2010). The German labor market response in the world recession—de-mystifying a miracle. J. Lab. Mark. Res..

[CR23] OECD (2010). OECD economic surveys: Luxembourg.

[CR24] Oi WY (1962). Labor as a Quasi-fixed factor. J. Pol. Econ..

[CR25] STATEC. Recours massif au chômage partiel en 2009, *Note de conjoncture. n°2*–*09*: Encadré: 64–71 (2009)

[CR26] Van Audenrode M (1994). Short-time compensation, job security, and employment contracts: evidence from selected OECD countries. J. Pol. Econ..

